# A case of *Massilia timonae* identified during time-lapse ART cycles in a pre-implantation embryo

**DOI:** 10.5935/1518-0557.20240061

**Published:** 2024

**Authors:** Alina S. Shurygina, Olga S. Guseva, Oksana V. Shurygina, Artem V. Lyamin

**Affiliations:** 1 Professional Center for Education and Research in Genetic and Laboratory Technologies / Federal State Budgetary Educational Institution of Higher Education Samara State Medical University of the Ministry of Healthcare of the Russian Federation. Samara, Russian Federation

**Keywords:** Massilia timonae, intra cytoplasmic sperm injection, time-lapse, infertility

## Abstract

This article reports a case of *Massilia timonae*, an understudied Gram-negative rod, in the culture media of an embryo produced by intracytoplasmic sperm injection into the oocyte cytoplasm. Cultivation was monitored with a time-lapse technology. Microbial contamination can cause embryo developmental arrest.

## INTRODUCTION

Bacterial contamination of embryos and culture media *in vitro* is a rare phenomenon. It can damage and cause the loss of cultivated oocytes and embryos. According to published data, the infection rate is between 0.22% and 0.86% (Shu *et al.*, 2016; Vaduva *et al.*, 2022).

*Massilia timonae* is an underinvestigated aerobic Gram-negative rod belonging to the *Massilia genus* of the *Oxalobacteraceae* family. It was first isolated and described in 1998 in the blood of a young man with meningoencephalitis and generalized variable immune deficiency (La Scola *et al.*, 1998). *Massilia spp.* are ubiquitous in the environment and found in plants, soil, and drinking water (Miess *et al.*, 2020). There are few reports of cases or series describing human infection by *Massilia timonae*, and the clinical significance of this pathogen is still unclear (Huang *et al.*, 2018). This report describes a case of contamination by *Massilia timonae* of the culture media used in an embryo produced by intracytoplasmic sperm injection (ICSI) into the oocyte cytoplasm. The bacterial contamination of one of several embryos was identified during cultivation using a time-lapse embryo development monitoring technique.

## CASE HISTORY

In 2023, a 33-year-old woman (body mass index 26 kg/m^2^) sought care at an *in vitro* fertilization (IVF) clinic after failing to conceive for seven years despite regular sexual activity without contraceptives. Her medical history included an abortion in 2015 at a gestational age of five weeks while she was with another partner, polypectomy, pseudo-erosion, chronic endometritis, and hypothyroidism. Her spouse was 37 years old and had a history of tuberculosis, diagnosed in 2019 and treated with surgery and chemotherapy.

The woman’s endocrine profile was as follows: follicle-stimulating hormone 7.15mIU/mL, luteinizing hormone 3.9mIU/mL, anti-Müllerian hormone 2.17ng/mL, thyroid-stimulating hormone 2.32mIU/mL, prolactin 488ng/mL, estradiol 118pg/mL, and testosterone 1.36nmol/L. Vaginal and cervical smears found deoxyribonucleic acid (DNA) of *Ureaplasma sp.* and *CMV* (detected by real-time polymerase chain reaction, PCR) and up to 30 white blood cells. The spouse’s semen analysis showed 900,000 total sperm cells, with 11% motility and indefinite morphology (due to insufficient cell concentration). Real-time PCR of the male genitourinary tract flora detected no DNA of pathogens. The complaints, medical history, examination data, expert opinions, laboratory tests, and instrumental diagnostics indicated a clinical diagnosis as follows: female infertility associated with male factors; oligospermia; hypothyroidism, manageable; vaginitis, unspecified. Taking into account the failure to conceive naturally, the patient was invited to join an infertility treatment program in which she was prescribed ICSI followed by embryo transfer after a cycle of Polygynax vaginal capsules (Catalent France Beinheim SA, France), doxycycline, and valacyclovir.

After successful therapy, she underwent multifollicular ovarian stimulation based on a protocol with LHRH antagonists, which was completed uneventfully.

A sample of her spouse’s sperm was collected by masturbation into a sterile container on the day ovary paracentesis was performed. No significant alterations were observed in the semen parameters on the paracentesis day compared to the previous analysis. In this study, the sperm was handled using Sil-Select Plus media (FertiPro, Belgium), which contains gentamycin.

On day 15, 16 follicles were picked up, and 16 oocytes were obtained. ICSI produced six embryos. The embryos were cultivated pre-implantation using G-TL universal media (Virtolife, Sweden) under OVOIL until day 5. The culture media for the fertilized oocytes and embryos contained gentamycin. The cultivation was performed in an EmbryoScope+ incubator (Vitrolife, Sweden).

**Figure 1:**
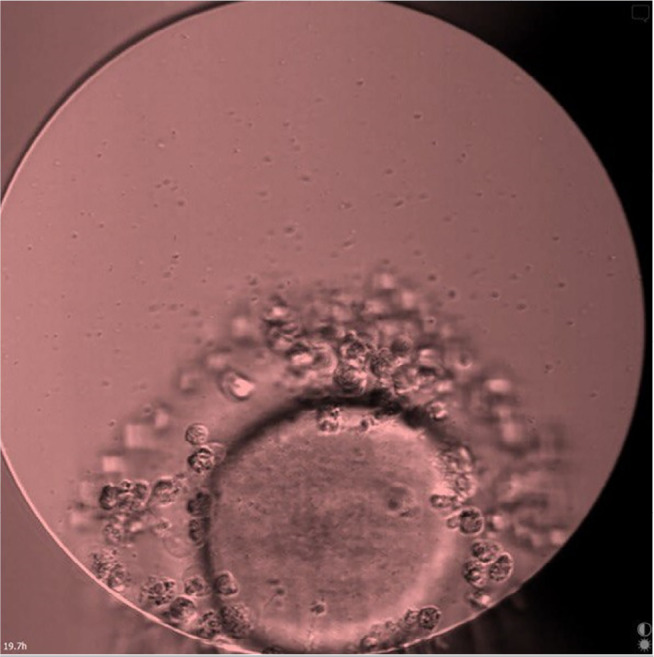
An embryo in the contaminated culture 19 hours after ICSI (×200).

On day 1 of cultivation, 19 hours after ICSI, bacterial content was found in one of six embryo wells during fertilization assessment (Figure 1).

The other five wells had no signs of infection. Four embryos, including the infected one, had their development arrested during cultivation. Two good-quality embryos (with no infection signs) were frozen. The media used to cultivate the arrested embryos were collected and submitted to a microbiological investigation. Rinse blanks were collected from the laboratory working areas for microbial contamination assessment. Sample processing, inoculation, and incubation were performed in anaerobic conditions, which were maintained using a Bactron 300-2 anaerobic chamber (Sheldon Manufacturing Inc., USA). Colony growth was observed on the anaerobic agar (HiMedia, India). The isolated microorganisms were identified with MALDI-ToF mass spectrometry performed on a Microflex LT instrument (Bruker, USA) in the Standard mode. The tests revealed contamination by *Massilia timonae*. No microorganisms were found in the samples from the laboratory working areas.

## DISCUSSION

The probability of microbial contamination in human embryo cultures is generally minimal. ART laboratories are qualified as class A clean rooms. However, the possibility of microbial contamination still exists. In most cases, the sources are human sperm or follicular fluid. Another potential source is the laboratory staff (Vaduva *et al.*, 2022; Zheng *et al.*, 2023). In our study, rinse blanks from the working areas tested negative for contamination. The bacterium was only found in a single plate well with cultured embryos, which probably caused the embryo’s developmental arrest. The follicular fluid might have caused the contamination, as each follicle was picked up separately into a clean, dry tube. It is unlikely that sperm was the source of contamination since the sperm cells were injected into the oocytes using ICSI instruments instead of the conventional addition of a suspension. Besides, bacterial contamination is generally reported in IVF rather than ICSI cycles (Shu *et al.*, 2016; Borges *et al.*, 2020). Nonetheless, in our case, contamination occurred during an ICSI cycle.

The media used in fertilization and embryo cultivation contain antibiotics. However, the oocyte is surrounded by the zona pellucida, which has a porous, mesh-like structure. Removing microorganisms from it by rinsing and culturing embryos in such media may be challenging (Li *et al.*, 2022). Many bacteria possess antibiotic resistance, and standard media may not produce the desired effect. Instead, it preserves the bacteria and promotes their growth (Zheng *et al.*, 2023).

In our case, the microorganism that contaminated the embryo culture media was *Massilia timonae*, a pathogen rarely reported in cases affecting humans. However, a report indicated that the pathogen was linked to a septic abortion (Ali *et al.*, 2022). Therefore, contamination by *Massilia timonae* in an IVF cycle requires close attention. This bacterium may affect both the *in vitro* embryo development and the pregnancy outcome after embryo transfer. The pathogen may enter the uterus during embryo transfer and cause infection, which may cause pregnancy loss and harm the patient’s health. The optimal duration of treatment for *Massilia timonae* infection is still unknown (Ali *et al.*, 2022).

Considering the growing number of ART-based infertility treatment cycles, this issue cannot be underestimated (Zheng *et al.*, 2023). Cases of contamination by this pathogen are likely underreported, which explains the low number of cases discussed in the literature.

In conclusion, future studies should focus on the microbiological analysis methods used to examine the biological material obtained from couples undergoing IVF. This will help reduce the potential negative effects of adverse factors on embryo development.
